# Systematic research is needed on the potential effects of lifelong technology experience on cognition: a mini-review and recommendations

**DOI:** 10.3389/fpsyg.2024.1335864

**Published:** 2024-02-16

**Authors:** Monique E. Beaudoin, Kelly M. Jones, Bernadette Jerome, David Martinez, Tim George, Nick B. Pandža

**Affiliations:** Applied Research Laboratory for Intelligence and Security, University of Maryland, College Park, MD, United States

**Keywords:** technology, cognition, adaptability, creativity, decision-making, functional fixedness

## Abstract

Digital technology now occupies a fundamental space in human life. Increasingly sophisticated access to information and social interactions has enabled a sort of offloading of many aspects of cognition, and for many people, this technology use has been lifelong. While the global development of technologies advances exponentially as part of the Fourth Industrial Revolution, researchers have not yet fully characterized the human effects of this technology-centric revolution at the same pace. In this mini-review, we consider three important higher-level cognitive functions: creativity, adaptability, and decision-making, and discuss their potential relationship to lifelong digital technology experience, which here includes both passive exposure and active use of electronic devices. We then articulate the gaps in related literature and knowledge, and outline general considerations, suggestions, and challenges for future research avenues. In general, we found that prior research has investigated uses of specific technology products on lower-level cognition (e.g., how does the use of online search engines affect memory?), but there is a lack of research assessing the overall effects of technology experience on cognitive functioning, particularly complex cognition.

## 1 Introduction

Stop us if you've heard these before: “Is your cell phone causing your brain fog?” (Rideout and Jones, 2023); “Social media could be harming your child's attention span” (Reed, 2022); or “Subtle ways technology is making humanity worse” (Greenwood, 2019). Occasionally, one might see the inverse headline: “Opinion: How technology is teaching kids to care about the world and each other” (Alrubail, 2018). These and other sensationalist headlines are designed to play into algorithmically-driven engagement on the very platforms being decried. Articles are not always op-eds; these types of headlines often come from press coverage of scientific research (e.g., Ophira et al., 2009).

Fundamental shifts in usage of screen-based interactive technology have occurred in the last 20–30 years. As the pace of innovation increases while entry costs decrease, increased connectivity and technology use occur concomitantly. Technologies are becoming more permanently integrated into everyday life, driving further adoption and changes to processes, societal norms, and standards of engagement.

Existing research on the psychological effects of technology use is often limited to studying the social and/or academic impacts of social media use (a limited definition of “technology”) and often in children and adolescents (e.g., Firth et al., 2019; Gottschalk, 2019; Meshi et al., 2019; Oswald et al., 2020). Such studies leverage both experimental manipulations of a particular technology use (e.g., the effects of laptop note taking on lecture memory; see Mueller and Oppenheimer, 2014) and correlational studies predicting outcomes based on individual differences in specific technology behavior (e.g., the correlation between excessive internet use and social anxiety; see Weinstein et al., 2015). In contrast, research encompassing the cumulative effects of technology use on cognition over an individual lifespan is limited (cf. Kamin and Lang, 2020). An often-cited anecdotal example is that the generations raised using GPS instead of paper maps have lost (or never gained) the ability to navigate without GPS—but is this accurate (cf. Dahmani and Bohbot, 2020)? Are there also benefits to be had by freeing up cognitive resources for other tasks? What changes in cognitive capabilities can we anticipate in the era of ChatGPT and the widespread adoption of generative AI tools as task assistants?

It is difficult to identify the effects of a specific technology on cognitive functions with precision; it is even harder to define and measure the effects of technology use cumulatively over a lifespan. However, the question of lifelong impacts of technology use on cognition is an important one that has been neglected, and it requires novel methods of inquiry.

In this mini-review, we consider three important higher-level cognitive functions: creativity, adaptability, and decision-making. Below, we provide overviews of each construct and their measurement and discuss their potential relationship to lifelong digital *technology experience*. For this mini-review, we use “technology experience” broadly to refer to passive and active use of electronic devices across time and at a range of proficiency levels. We then articulate the gaps in present knowledge and outline general considerations, suggestions, and challenges for future research avenues.

## 2 Methodology

### 2.1 Literature searches

The authors performed literature searches through publicly available peer-reviewed journals and databases (e.g., Google Scholar). Keywords used for searches were standardized by a team of subject matter experts, with the goal of identifying prior research related to measuring technology reliance/experience and cognitive testing, particularly any measures of effects of technology use over a lifetime. Given that the scope of potentially affected cognitive functions is broad, the team focused on three categories for this initial search: (1) creativity, (2) adaptability, and (3) decision-making. All three of these have a wealth of prior research and measures. Some prior research has begun to examine effects of technology use on more basic attention and memory processes (Storm and Soares, 2022). In contrast, our three foci represent higher-level cognitive processes with broad importance for real-world contexts. Moreover, we note that the literature has partly focused on factors that may improve creativity (e.g., Scott et al., 2004), adaptability (e.g., Kroneisen et al., 2021) and decision-making (e.g., Fukukura et al., 2013; Schacter et al., 2017), suggesting that these constructs are susceptible to change.

### 2.2 Defining “technology”

Next, the team determined how “technology” would be defined for our investigation, as well as which sorts of technology are in-scope when considering measurements of *technology experience*. Merriam-Webster defines technology as “the practical application of knowledge especially in a particular area” (n.d.),^1^ a specification so broad as to include even the pencil as an example of communications technology. We defined technologies of interest for this work as hardware and software that have screen-based interaction(s), making simultaneous use of information retrieval and often direct interaction (see Table 1).

**Table 1:** In- and out-of-scope technologies, based upon screen-based interactions.

**In-scope technologies**	**Out-of-scope technologies**
1. Mobile phones 2. Desktop computers 3. Immersive technology a. Virtual reality b. Augmented reality 4. Videogames a. All types, including builder games, shooter games, etc. 5. Geocaching 6. Social media 7. Messaging apps 8. Driverless car technology 9. Navigation devices, commonly referred to as GPS (Global Positioning Service) e. Note that GPS is often accessed through mobile phones	1. Land-line telephones 2. e-Readers 3. Screen/software-based decision support tools

## 3 Literature review

### 3.1 Creativity

Creativity is typically defined as the production of novel and useful ideas (Runco and Jaeger, 2012) ranging from major breakthroughs to everyday creative acts, like using a shoe as a planter (Stein, 1987; Merrotsy, 2013). Creativity as a *process* is complex and not yet fully understood. Consequently, several theories attempt to capture some aspect of the creative process. For example, one theory contends that creativity involves the association of disparate ideas (Mednick, 1962); another posits that both *divergent* thinking (generating multiple possible ideas) and *convergent* thinking (selecting one best solution) are necessary (Guilford, 1968). A third conception is that creativity involves restructuring, which refers to altering a mental representation in a novel way (Ohlsson, 1984). In the example above, one's perspective of a shoe is altered from footwear to a container. Additionally, current theorizing surrounds the contributions of executive functions to these processes (Benedek and Jauk, 2019). Technology influences the process of creativity, changing and broadening access to information and ideas and providing new tools and platforms for creative exploration (Wingström et al., 2022; Obeid and Demirkan, 2023; Rafner et al., 2023).

#### 3.1.1 Measures of creativity

**Figure 1:**
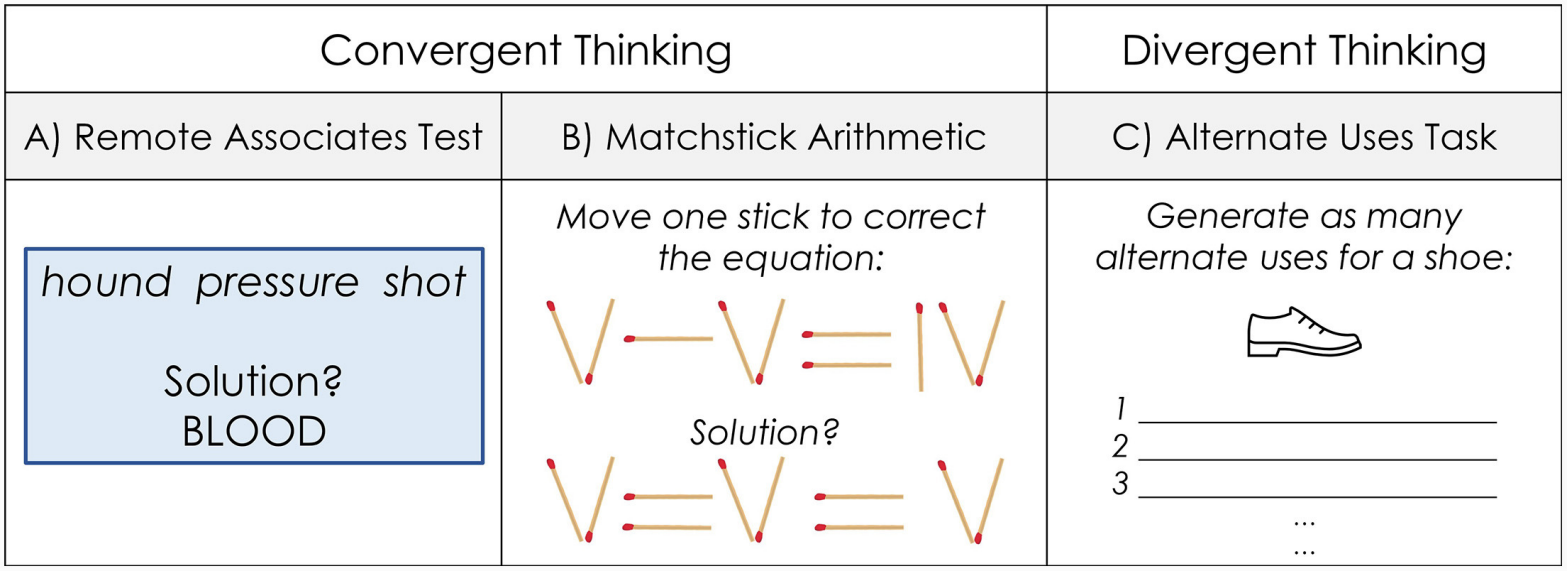
Example tasks used for the measurement of creativity.

Given the complexity of the creative process, there are a variety of approaches to measuring creativity. We describe a relevant subset of these here. One type of measures employs problems or puzzles with non-obvious solutions. These may exploit *convergent thinking*, because they typically have a single solution. For example, the remote associates test (RAT) is based on the associative theory described above (Mednick, 1962). Participants are given three words and generate a fourth word that is associated in some way with the other three (see Figure 1A). Additionally, there are so-called “insight” problems which resemble brainteasers. In matchstick arithmetic, one must make a mathematical statement true by moving a single matchstick (see Figure 1B). These problems often create an initial impasse, followed by a sudden emergent solution (an “aha!” moment). This contrasts with more incremental problem solving (Metcalfe and Wiebe, 1987). These problems also require restructuring: in matchstick arithmetic, one must restructure the problem as a series of lines rather than as fixed numbers and operations. It should be noted that insight experiences can occur with RAT problems as well (Bowden et al., 2005).

In contrast, other tasks prompt people to generate multiple possible ideas, sometimes called *divergent thinking* tasks. The Torrance Test of Creative Thinking inspired many of these tasks, such as the alternate uses task (AUT) (Goff and Torrance, 2002). In the AUT, one generates as many creative uses as possible for a common object (see Figure 1C). Ideas are typically assessed for quantity, flexibility, and novelty (Reiter-Palmon et al., 2019).

#### 3.1.2 Effects of technology experience on creativity

Some research has attempted to investigate interactions between technology and creativity. However, much of this research investigates the impact of specific uses of technology on creativity, such as: digital technology products improving student creativity (Tang et al., 2022), the beneficial effects of videogame playing (Jackson et al., 2012), and potentially negative effects of social media (Upshaw et al., 2022) and smartphone use (Olson et al., 2022). These studies are thus inherently limited in the generalizability of their findings, and much less is known about the relationship between overall lifelong experience with digital technology and creative cognition.

### 3.2 Adaptability

Adaptability here is an umbrella term referring to the ability to recognize and adjust one's behavior in a dynamic environment, at the moment of need. Adaptability includes the measurable constructs of cognitive flexibility; attention switching; task switching; inhibition control; response and representational flexibility; and overcoming functional fixedness. Technology can certainly support adaptability, for example by providing real-time information about the environment; however, the question posed here is whether lifelong technology experience can affect cognitive processes underlying adaptability. For example, does the constant need to adapt to new technologies and interfaces exercise and therefore improve our adaptability?

#### 3.2.1 Measures of adaptability

Task switching is the ability to shift attention and move between tasks (Jersild, 1927; Wylie and Allport, 2000). It is often assessed through “switch cost,” in which response time or accuracy in one task is compared to alternating between two tasks with different goals or rules, requiring inhibitory control of the prior task set (Arrington and Logan, 2004). Other related measures include the Wisconsin Card Sorting Task (Coulacoglou and Saklofske, 2017) and the Stroop Task (Stroop, 1935).

Functional fixedness is a cognitive bias that limits use of objects to their intended use—a hammer to pound in nails (Duncker, 1945). Cognitive flexibility allows us to imagine and use objects in novel or unusual ways. Thus, overcoming functional fixedness can be part of problem solving or creativity when faced with a challenge that requires a unique, unexpected, or unconventional solution (Adamson, 1952) in either lab-based/artificial (German and Barrett, 2005) or real-world settings (McCaffrey, 2012).

Adaptability and creativity are overlapping constructs with at least one difference: adaptability requires a behavior change to be useful, while for a change to be considered creative, it must *also* be novel. Thus, all measures of creativity can be seen as partly measuring adaptability, though not all measures of adaptability are measures of creativity (e.g., the Stroop task).

#### 3.2.2 Effects of technology experience on adaptability

There is limited evidence of the effect of technology experience on cognitive flexibility, and research tends to be limited to comparing like tasks—for example, switching between email and social media. There is evidence that switching between two similar tasks both requiring heavy cognitive loads (such as switching between meetings on different topics) depletes mental and physical resources (Arrington and Logan, 2004), as does switching between low-load tasks that are dissimilar from each other. There is minimal research investigating the effects of switching between technological to non-technological tasks or environments, and research that does exist largely uses a single technology of interest, such as voice calls on mobile phones (e.g., Abramson et al., 2009).

Evidence suggests heavy media multitaskers develop the skills to rapidly switch between tasks without loss of focus when switching between tasks on technological devices, though results are mixed (Rosen et al., 2011; Alzahabi and Becker, 2013). So-called “digital natives” who grew up with access and exposure to technology from an early age tend to show high levels of adaptability with new and advancing technologies (Barak, 2018). However, evidence suggests that a period of smartphone separation can cause increased switch costs (Hartanto and Yang, 2016). The mix of evidence regarding the effects of technology use on adaptability constructs requires further research across differing mediums, tasks, and populations.

### 3.3 Decision-making

Decision-making refers to processes involved in making choices among alternatives, occurring in both a conscious and subconscious processing capacity (van Gaal et al., 2012). Decisions can be simple with well-defined options (“Should I purchase product A or B?”) or require highly complex hierarchical reasoning (“Which course of treatment should I give my patient based on their changing symptoms?”).

There are various models that exist to describe the flow of decision-making or components thereof (e.g., attention, emotion, memory). Early models made assumptions about people behaving rationally during decision-making to maximize utility (Edwards, 1954). Later research revealed that decision-making is informed by reasoning, and reasoning is notoriously biased due to a tendency to rely on heuristics (Kahneman, 2003). However, this research was based on relatively simple paradigms involving simultaneous presentation of clearly described alternatives. In the real world, decision-making unfolds in changing, uncertain, and complex environments. Recent conceptualizations are based on closed-loop models where decision-making is an iterative and dynamic cognitive process in which one's choices are influenced by the outcome of prior decisions, current goals and context, prior experience, knowledge and biases (Gonzalez, 2017). As decision-making is influenced by the information available, and technology affects information access, it stands to reason that technology experience may alter decision-making behaviors and capabilities (e.g., an inability to make decisions without the aid of Google).

#### 3.3.1 Measures of decision-making

Several validated tests exist to measure various components of decision-making. The Adult Decision-Making Competence scale (ADMC) assesses accuracy and consistency in decisions (Parker et al., 2018). The Iowa Gambling Task (IGT) is a psychological test that simulates real-life decision-making in addition to measuring impaired and risky decision-making (Bechara et al., 1994). Prezenski et al. (2017) suggested that the Wisconsin Card Sorting Test (typically considered a flexibility measure) can serve as a laboratory-based dynamic decision-making measure. Dynamic decision-making accounts for preferences changing over time, in addition to decisions depending on previous feedback from a potentially changing environment (Busemeyer and Townsend, 1993).

#### 3.3.2 Effects of technology experience on decision-making

There is limited research on the effects of technology experience on decision-making. Meshi et al. (2019) found that participants who reported excessive Facebook usage scored lower in a portion of the Iowa Gambling Task. Additionally, emerging AI tools may affect human decision-making. Recent work by Buçinca et al. (2021) suggests that AI-assisted decision-making may lead to overreliance on AI suggestions, but that *cognitive forcing functions*—interventions designed to prompt more deliberate, analytic thinking—can reduce this overreliance. Other related work has suggested that proliferation of information online can exploit people's susceptibility to misinformation (Pennycook and Rand, 2021). Given its increasingly sophisticated role in aiding human cognition, the overall effects of technology on how we make complex decisions is an area in need of exploration.

## 4 Discussion

This mini-review has provided an overview of the limited research on the effects of lifelong technology experience on three important cognitive capabilities. Results from the few extant studies are mixed or even contradicting in terms of whether the observed effects are positive or negative. In addition, most prior work has focused on the social or academic impacts of specific technology use (particularly social media use) and often in children and young adults (e.g., Firth et al., 2019; Gottschalk, 2019; Meshi et al., 2019; Oswald et al., 2020). While this is valuable and necessary work, it does not encompass the full range of potential effects of technology experience, including the cumulative use of (ever-changing) technologies over a lifetime. Nor does it answer the fundamental question of how the permanent integration of screen-based technologies (or the potential impacts of the next technological innovations) into day-to-day living is changing the ways we think about (and interact with) our world. Therefore, we contend that further research is needed across disciplines to develop and validate new methodologies focused broadly on impacts of lifelong technology experience on cognitive processes. Below, we lay out the following suggestions, considerations, and likely challenges for future research—broadly speaking, in two categories: issues of measurement, and issues of design and causality.

### 4.1 Issues of measurement

We recommend the development of validated assessment(s) of lifelong technology experience. In our literature review, we found that measures of “technology use” generally had one or more of the following limitations: (1) they were highly bespoke to each study and used myriad definitions of technology; (2) measures of effects of technology (i.e., the dependent variables) were likewise bespoke to each study, and generally used a very narrow (often mental health focused) type of effect; (3) did not comprehensively measure technology use across mediums or lifelong usage; (4) were subjective self-report, with very few (more objective) behavioral measures and; (5) were implemented in convenience samples of students or otherwise white, educated, industrialized, rich, and Democratic populations (WEIRD; Henrich et al., 2010) (see also Wilmer et al., 2017; Ellis, 2019; Firth et al., 2019). These issues raise questions about the validity and generalizability of existing measures.

To answer the questions posed in this article, researchers should develop and validate measure(s) of technology experience across the lifespan that encompass a wider range of technologies, their uses, and their effects - particularly on cognition. Additionally, because of the high average level of technology use in Western societies, future measures will need to sensitively capture variability in technology experience across different socioeconomic and cultural contexts, if intended for broader usage. To examine the behaviors of interest more meaningfully, we recommend studies using broader sampling methods and ecologically-relevant measures. Subsequent research should investigate the replicability of novel measures to examine their validity and generalizability across various populations and the lifespan.

### 4.2 Issues of design and causality

Given the state of the research presented above, more targeted and replicable research is needed to understand how cognitive attributes may vary as a function of lifelong technology experience. One could first test for significant correlations (e.g., Dahmani and Bohbot, 2020). Of note, interpretations of causality will be difficult and will require statistical control of multiple variables (see Rohrer, 2018) and mediational analyses. Therefore, longitudinal studies of deviations from baseline across multiple time points in diverse populations are also critical and would necessitate the perspectives of developmental psychologists. Other research designs (e.g., quasi-experimental and cross-sectional) could be used for comparing groups with systematic differences in technology experience (e.g., German and Barrett, 2005). Given the interconnected nature of these constructs with daily living and the issues with assessing causality noted above, this will present a challenge. However, with multiple converging lines of evidence and use of open-science practices with well-defined and replicable methodologies, this avenue of research will advance our understanding of technology's relationship to cognition.

### 4.3 Future technological evolution

Finally, we note that research in this area needs to evolve as technology advances. For example, a current topic of research interest is the potential effect of AI on the workforce. How will people perform tasks that formerly required purely human engagement (e.g., writing articles) when AI can accomplish those tasks instead? What happens to cognitive abilities not regularly used as future technologies emerge?

In conclusion, technological developments have occurred so rapidly in the past several decades that research regarding its effects on human cognition has not kept pace. Valuable experimental and correlational findings have emerged about specific technological tools (e.g., Google, Facebook) and their impacts on social or lower-order cognitive attributes (e.g., Firth et al., 2019); however, there is currently insufficient empirical research to characterize the effects of general technology experience over a lifetime, especially for higher-order cognition. We hope the considerations outlined in this review will help set the stage for future research.

## Author contributions

MB: Conceptualization, Project administration, Supervision, Writing—original draft, Writing—review & editing. KJ: Conceptualization, Supervision, Writing—original draft, Writing—review & editing. BJ: Writing—original draft, Writing—review & editing. DM: Writing—original draft, Writing—review & editing. TG: Writing—review & editing. NP: Conceptualization, Writing—original draft, Writing—review & editing.

## Footnotes

^1^Technology. In Merriam-Webster.com dictionary. Retrieved January 4, 2023, from https://www.merriam-webster.com/dictionary/technology.
